# A comprehensive standardized dataset of numerous pomegranate fruit diseases for deep learning

**DOI:** 10.1016/j.dib.2024.110284

**Published:** 2024-03-01

**Authors:** Pakruddin B․, Hemavathy R․

**Affiliations:** a Department of Computer Science & Engineering, R.V. College of Engineering, Visvesvaraya Technological University, Belagavi 590018, India; b Department of Computer Science & Engineering**,** Presidency University**,** Bengaluru 560064, India

**Keywords:** Pomegranate diseases, Dataset, Detection and classification

## Abstract

Pomegranate fruit disease detection and classification based on computer vision remains challenging because of various diseases, building the task of collecting or creating datasets is extremely difficult. The usage of machine learning and deep learning in farming has increased significantly in recent years. For developing precise and consistent machine learning models and reducing misclassification in real-time situations, efficient and clean datasets are a key obligation. The current pomegranate fruit diseases classification standardized and publicly accessible datasets for agriculture are not adequate to train the models efficiently. To address this issue, our primary goal of the current study is to create an image dataset of pomegranate fruits of numerous diseases that is ready to use and publicly available. We have composed 5 types of pomegranate fruit healthy and diseases from different places like Ballari, Bengaluru, Bagalakote, Etc. These images were taken from July to October 2023. The dataset contains 5099 pomegranate fruit images which are labeled and classified into 5 types: Healthy, Bacterial blight, Anthracnose, Cercospora fruit spot, and Alternaria fruit spot. The dataset comprises 5 folders entitled with corresponding diseases. This dataset might be useful for locating pomegranate diseases in other nations as well as increasing the production of pomegranate yield. This dataset is extremely useful for researchers of machine learning or deep learning in the field of agriculture for emerging computer vision applications.

Specifications Table

**Table:** 

Subject	Artificial Intelligence, Computer Vision, Data Science, Agriculture Engineering.
Specific subject area	Machine Learning and Deep Learning based Image Detection and Classification of Pomegranate Fruit Diseases.
Data format	Raw
Type of data	Table, Image, Figure
Data collection	Different types of pomegranate fruit diseases images were collected on different farms. The Redmi 9 Phone's high-resolution camera is used to capture the images of pomegranate. The novel images were in JPG format and the aspect ratio is 1:1 with a size of 3120 * 3120 pixels. These pomegranate images were taken in a sunny and cloudy environment on a farm. The images were taken from July to October 2023. These images were further categorized into five types: Healthy, Bacterial blight, Anthracnose, Cercospora fruit spot, and Alternaria fruit spot resulting in a total of 5099 images. The dataset was split into five folders, suitable for training deep learning models. The size of the folder for the dataset is 4.18GB and a RAR file was provided for appropriate downloading.
Data source location	Institution: Private Pomegranate Farm Land City/Town/Region: Ballari and Bengaluru, Karnataka State. Country: India Latitude, Longitude, and Altitude for collected samples: 14.82500, 76.60580, 610 meters. Date: 13/08/2023 Latitude, Longitude, and Altitude for collected samples: 13.16960, 77.57880, 915 meters. Date: 03/09/2023
Data accessibility	Repository name: Mendeley Data Data identification number: 10.17632/b6s2rkpmvh.1 Direct URL to data: https://data.mendeley.com/datasets/b6s2rkpmvh/1

## 1 Value of the Data

• We cover four major diseases that attack pomegranate fruits, affecting many pomegranate farms. Researchers can download the dataset, which is ready and accessible for public usage, and directly feed the data into deep learning algorithms.
• Pomegranate fruit disease datasets are valuable across agriculture, plant pathology, and data science, aiding in improving cultivation practices and promoting sustainable agriculture.
• Reusing pomegranate fruit disease datasets is essential for researchers to properly cite the source of the data and, if necessary, adhere to any usage terms or licensing agreements associated with the dataset. Additionally, researchers should consider sharing their findings and insights with the broader research community to contribute to advancements in agriculture and disease management.
• Pomegranate disease detection in agriculture has diverse applications, benefiting farmers with early disease detection, reduced chemical usage, crop monitoring, and quality assurance. The proposed dataset supports the development of advanced algorithms and techniques evaluation within the research community.

## 2 Background

Compiling the pomegranate fruit disease dataset aims to advance research and innovation in pomegranate agriculture, specifically focusing on disease identification and management. Leveraging machine learning, particularly deep learning, the dataset provides valuable insights for agriculture, plant pathology, and data science. The creation process involves meticulous image collection, annotation, and classification, emphasizing diversity in disease manifestations. This standardized dataset fosters collaboration, supports advanced algorithm development, and aims to enhance disease resistance and cultivation practices in pomegranate farming. As a standalone data article, it serves as a significant resource for researchers to benchmark and validate algorithms, contributing to the collective knowledge in the field and supporting ongoing and future research in pomegranate fruit disease identification and management.

## 3 Data Description

### 3.1 Description of major pomegranate fruit diseases and symptoms

Pomegranate is susceptible to several diseases that can adversely affect fruit quality and yield. Various agricultural specialists, horticulturists, and pomegranate plantation farmers have independently validated these diseases in pomegranate fruit images.

#### 3.1.1 Healthy

**Fig. 1:**
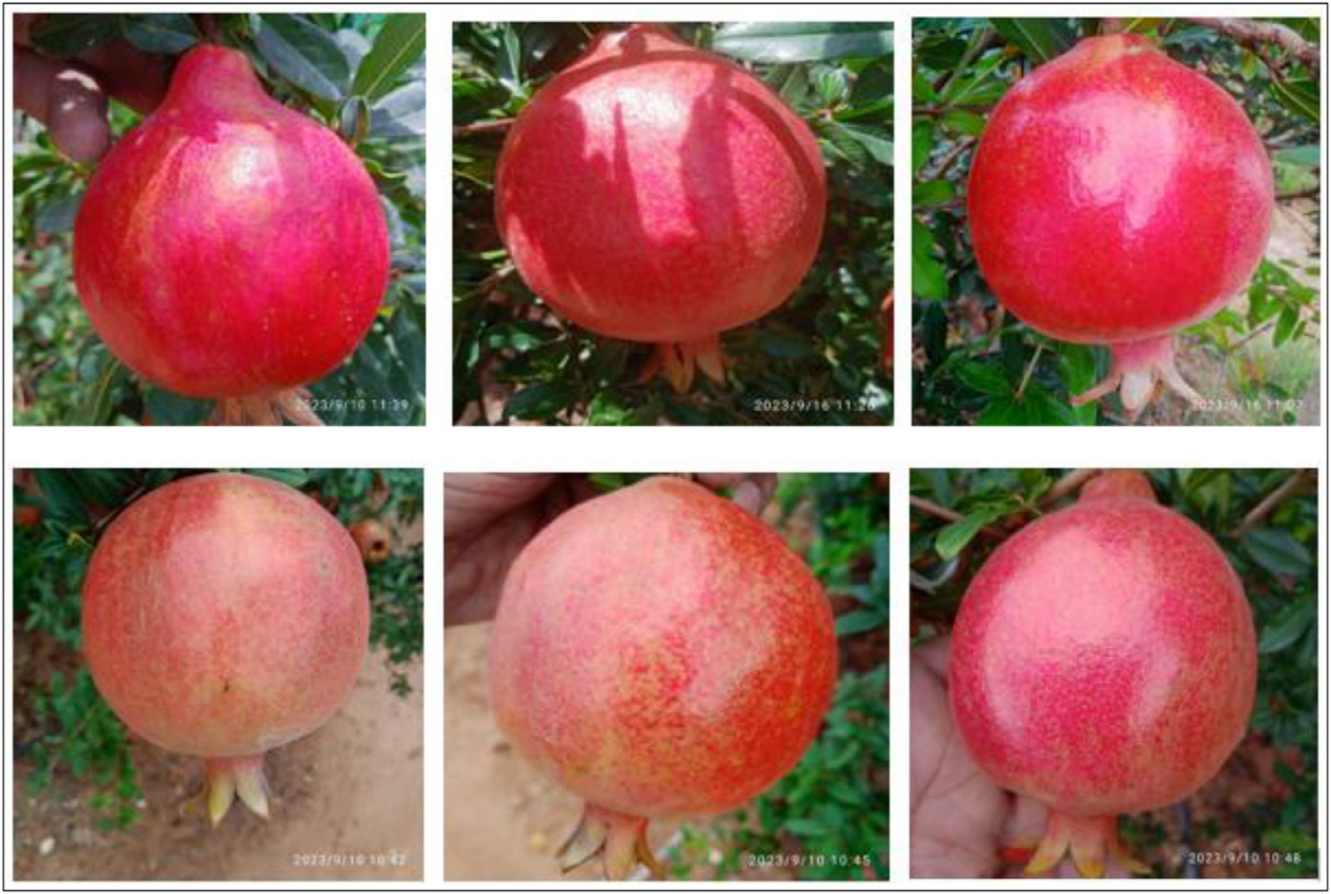
The Healthy pomegranate fruit.

A healthy pomegranate should feel firm and heavy for its size. Gently squeeze it to check for any soft spots or mushiness. Avoid fruits that are overly soft or have blemishes. The skin of a healthy pomegranate should be vibrant and have a rich, deep color, typically ranging from dark red to reddish-purple. However, the exact shade can vary depending on the variety. The skin should have a glossy appearance, which indicates that it is fresh and well-hydrated. Inspect the skin for any cracks, splits, or punctures. A healthy pomegranate should have smooth, unbroken skin, medium to large, and feel heavy in your hand compared to its size. Gently tap the pomegranate with your knuckles. It should produce a metallic, ringing sound. It should be well-formed and not shriveled or moldy. It should have a plump and round appearance, without any visible signs of withering or wrinkling. When you hold a pomegranate, it should feel evenly weighted, with no hollow or empty-feeling spots inside [1]. The sample images of healthy pomegranate fruits taken from the field are presented in Fig. 1.

#### 3.1.2 Bacterial blight

**Fig. 2:**
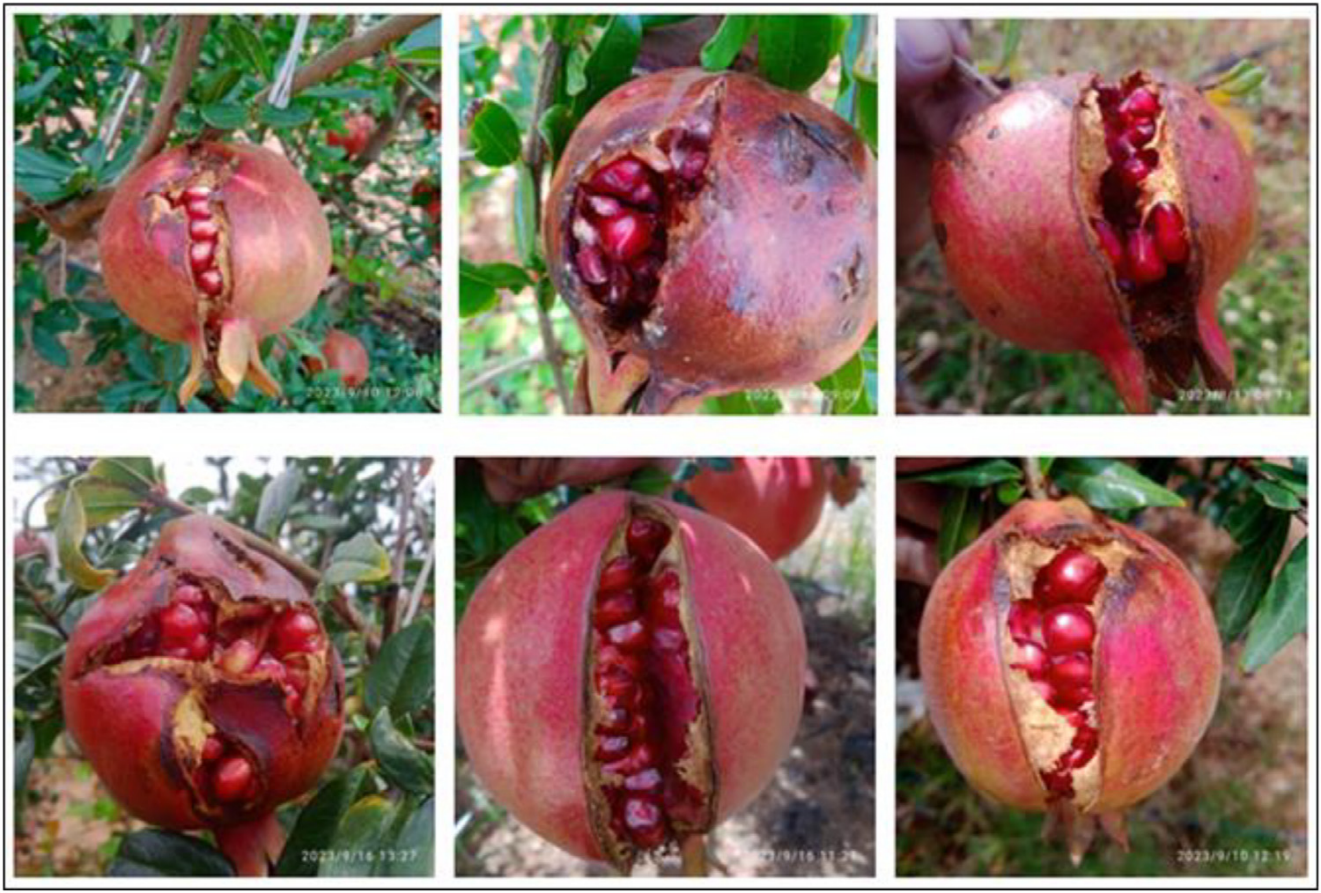
The Bacterial blight pomegranate fruit.

Bacterial blight is a common disease that affects pomegranate trees and their fruits. It can cause dark, sunken lesions on the surface of the fruit. These lesions may be circular or irregular in shape and are often surrounded by a reddish-brown or dark border. Infected fruit may exhibit a sticky exudate that oozes from the lesions. This exudate can be translucent or amber-colored. It may develop cracks or fissures around the lesions. These cracks can extend deep into the fruit and may promote secondary infections by other pathogens. In severe cases, pomegranate fruit affected by bacterial blight may prematurely drop from the tree before reaching maturity. This can result in significant yield losses. It may show discoloration, often with a water-soaked appearance, around the lesions. The color of the affected area can range from brown to black [2]. The sample images of Bacterial blight disease pomegranate fruits taken from the field are presented in Fig. 2.

#### 3.1.3 Anthracnose

**Fig. 3:**
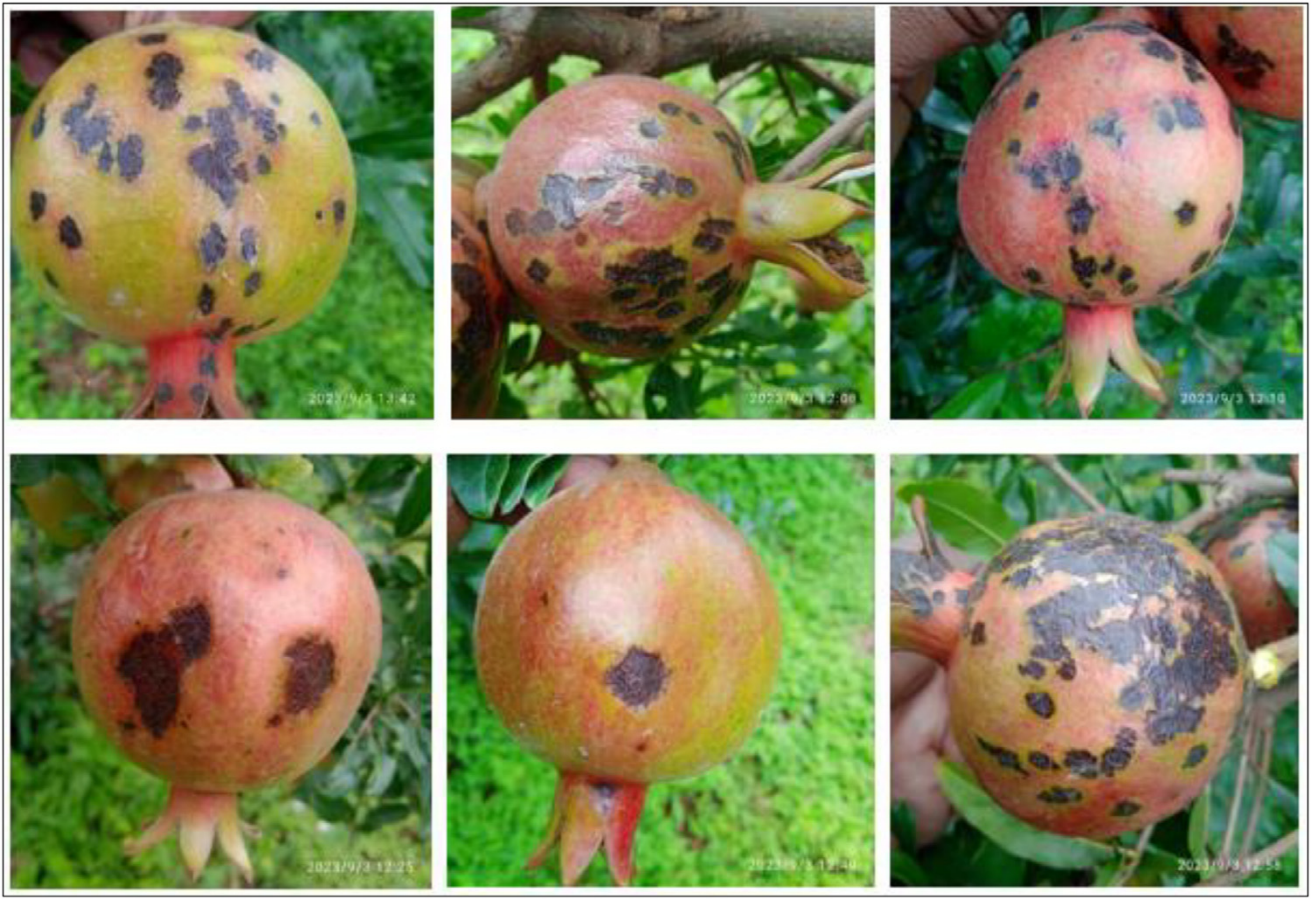
The Anthracnose pomegranate fruit.

Anthracnose disease signs on leaves, the calyx region, and fruits, there are tiny, regular to sporadic black specks that subsequently evolve into dark brown depressed patches, and infected fruit turn yellow. It is a common fungal disease that can affect pomegranate trees and their fruits. Anthracnose can cause circular or oval-shaped lesions on the surface of the fruit. These lesions may initially appear small and water-soaked, but they can enlarge over time. The lesions caused by anthracnose often become sunken as they progress. They may have a dark or brown center with a lighter-colored margin. Under humid conditions, pinkish or orange-colored spore masses may develop within the lesions. These spore masses are a characteristic feature of anthracnose. As the disease progresses, the infected fruit may start to rot. The rotting may be accompanied by a foul smell. Severe cases of anthracnose can lead to premature fruit drop, where the infected fruits fall from the tree before they fully mature [3]. The sample images of Anthracnose disease pomegranate fruits taken from the field are presented in Fig. 3.

#### 3.1.4 Cercospora fruit spot

**Fig. 4:**
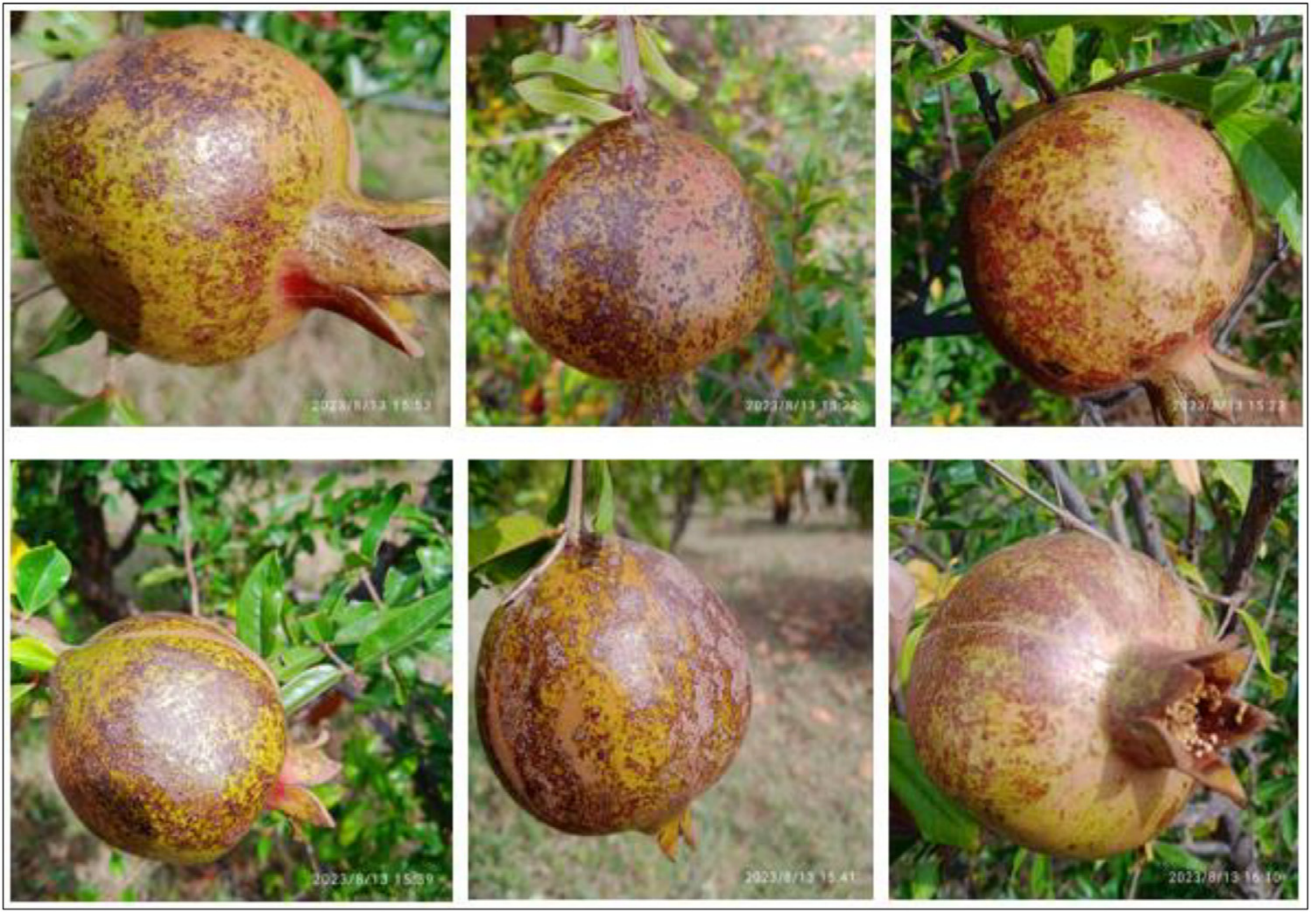
The Cercospora pomegranate fruit spot.

Cercospora fruit spot is a fungal disease that affects pomegranate fruit. It is caused by the pathogen Cercospora spp. and can lead to significant yield losses if not managed properly. The initial symptoms appear as small, circular to irregular spots on the fruit surface. These spots are usually brown or dark brown. Over time, the lesions tend to enlarge and may merge, forming larger irregular-shaped spots. The affected areas may also become sunken or depressed. As the disease progresses, the color of the lesions may change to gray or black, with a dark brown border. The affected fruit may develop an overall dull appearance. In severe cases, the fruit surface may crack or split near the lesions, providing entry points for secondary pathogens or decay-causing organisms. Infected fruits may prematurely drop from the tree before reaching maturity. This can result in significant yield reduction [4]. The sample images of Cercospora fruit spot disease pomegranate fruits taken from the field are presented in Fig. 4.

#### 3.1.5 Alternaria fruit spot

**Fig. 5:**
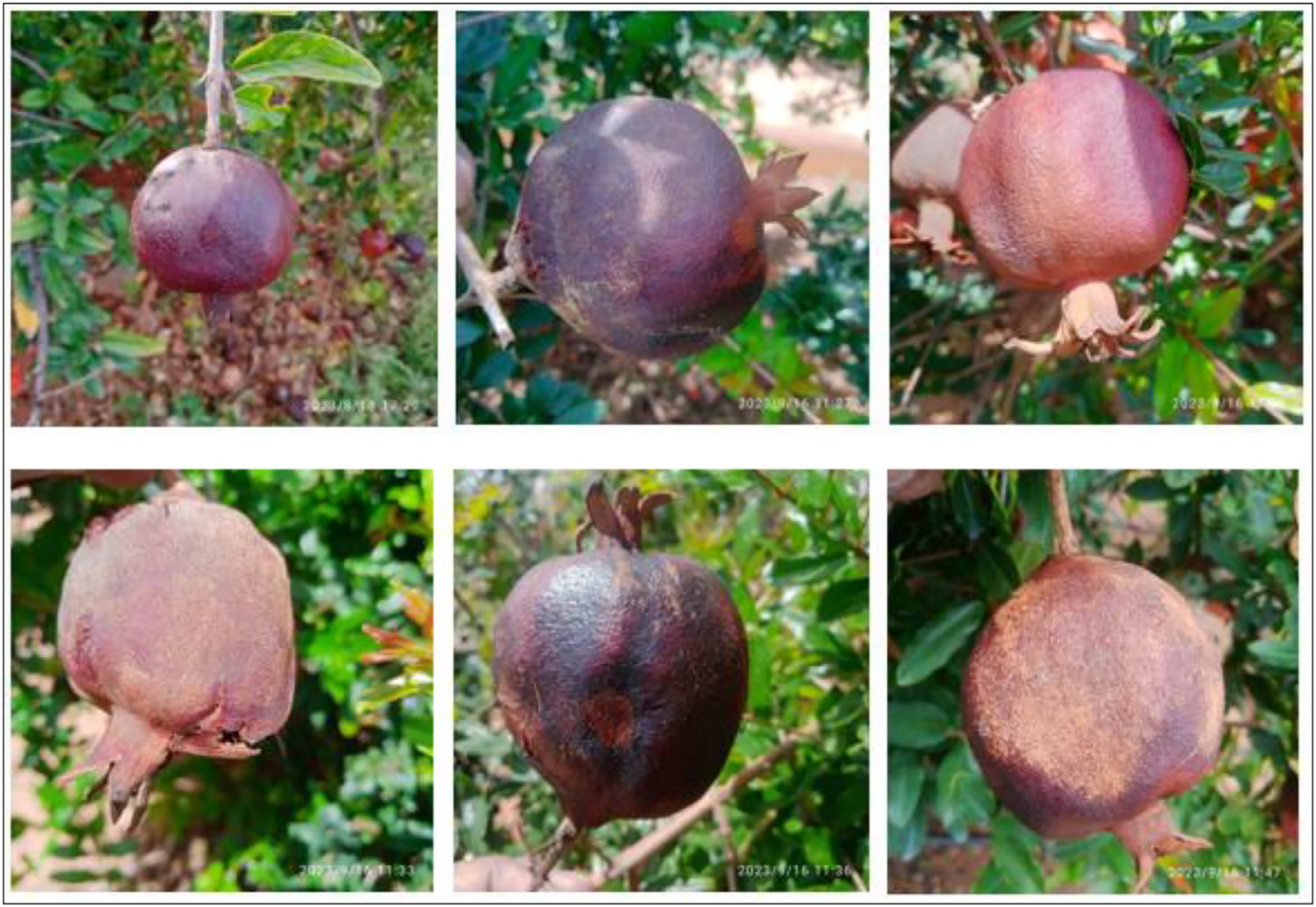
The Alternaria pomegranate fruit spot.

Alternaria fruit spot, caused by the fungus Alternaria alternata, is a common disease that affects pomegranate fruit. It primarily occurs in warm and humid conditions. The disease initially appears as small, brown, or black circular spots on the surface of the fruit. These spots may be slightly sunken and have a dark margin. As the disease progresses, the spots may enlarge and merge, forming larger irregularly shaped lesions. The affected areas may become soft and sunken. The lesions often turn dark brown or black as they mature. The center of the lesions may appear dry and corky, while the surrounding area remains slightly sunken. Under favourable conditions, the lesions may develop a velvety black appearance due to the presence of fungal spores. These spores are responsible for the spread of the disease. It's worth noting that the symptoms of Alternaria fruit spot can vary depending on the severity of the infection and environmental conditions [5]. The sample images of Alternaria fruit spot disease pomegranate fruits taken from the field are presented in Fig. 5.

### 3.2 Significance of the dataset

The dataset serves as a crucial resource for accurately identifying and managing diseases affecting pomegranate fruits, aiding in the development of effective strategies for disease control. By providing a comprehensive collection of data, the dataset facilitates the development of algorithms for early disease detection, enabling timely intervention to mitigate the impact on pomegranate crops [6]. Researchers and practitioners in precision agriculture can leverage the dataset to enhance farming practices, ensuring targeted and efficient application of resources for disease prevention and control. The dataset supports research endeavors, fostering innovation in the fields of agriculture, plant pathology, and data science. It provides a foundation for exploring new methodologies and techniques.

### 3.3 Description of dataset folders

**Fig. 6:**
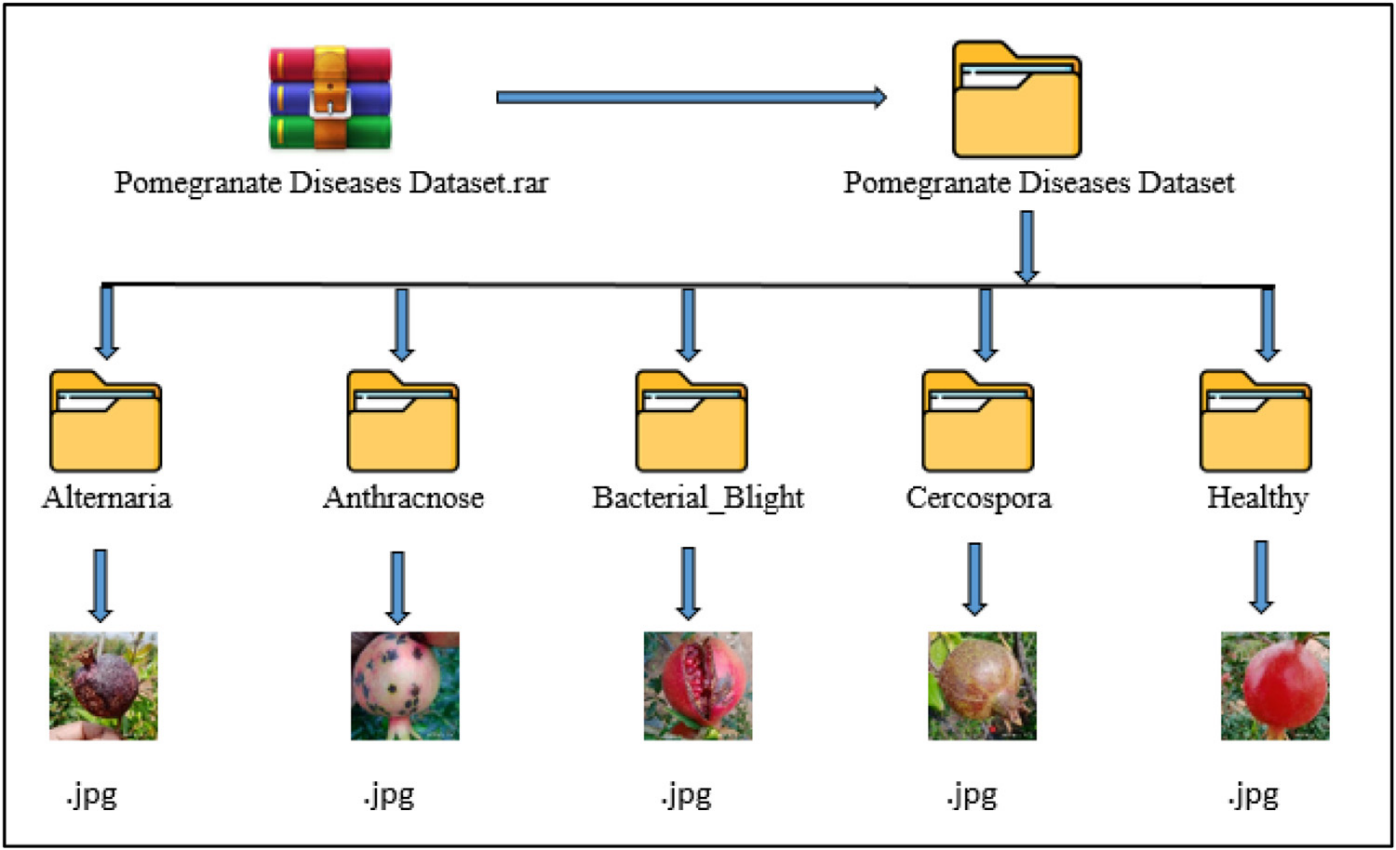
Structure of the dataset.

**Fig. 7:**
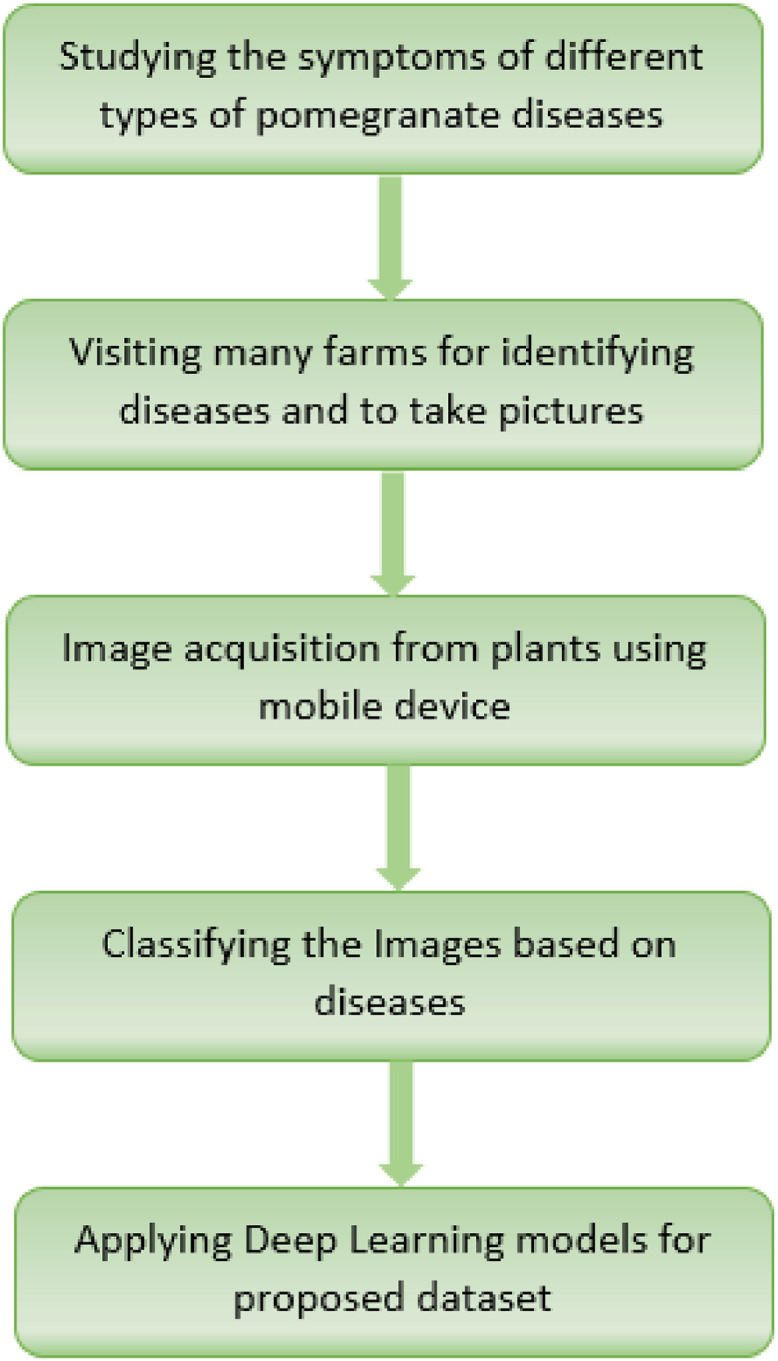
Workflow of the dataset preparation stages.

The description of dataset folders is shown in Fig. 6 and the workflow of the dataset preparation stages is shown in Fig. 7.

## 4 Experimental Design, Materials and Methods

### 4.1 Data gathering

India is now one of the world's top producers of pomegranates because of years of considerable development in pomegranate production. Pomegranates are grown in several states across the nation, with Karnataka, Gujarat, Maharashtra, and Andhra Pradesh functioning as the top producers. It is feasible to identify and categorize pomegranate fruit diseases based on images through the application of some computer vision and deep learning algorithms [7].

The proposed dataset contains images of various pomegranate fruit diseases gathered from various farms. Because these farms are affected by many diseases, we discussed with farm owners the issues and it is a major cause of crop failure to overcome these issues the study focuses on a specific fruit instead of many. We have collected 5 types of pomegranate fruit disease images including healthy from different places like Ballari, Bengaluru, Bagalakote, Etc. the pomegranate photographs were taken with the high-definition camera on the Redmi 9 Phone. The original photos were in the JPG file format, 1:1 aspect ratio, and 3120 × 3120 pixel size. These photos of pomegranates were taken on a farm in a sunny and cloudy environment from 9 am to 4 pm. The pictures were shot between July to October of 2023. A total of 5099 photos were produced after these photographs were then divided into five categories: Healthy, Bacterial blight, Anthracnose, Cercospora fruit spot, and Alternaria fruit spot. The dataset was divided into five folders that are ideal for deep learning model training. The dataset's folder is 4.18GB in size, and a RAR file has been made available for convenient downloading. We have also consulted and discussed with ICAR- Indian Institute of Horticultural Research, Bengaluru, to classify the respective images and fruits. The responsibility typically falls on a team of experts, including plant pathologists, researchers, and agricultural scientists. Their expertise in pomegranate fruit diseases enables us to accurately label images based on the observed symptoms and characteristics.

A decent and sufficient dataset can significantly improve the performance of machine learning algorithms such as Support Vector Machine, K-Nearest Neighbors, Artificial Neural Networks, etc., and deep learning algorithms such as Convolutional Neural Networks, Recurrent Neural Networks, etc., hence it is essential for their success. Deep learning algorithms excel at learning intricate patterns from data through the layers of a neural network. The hidden layers, in particular, act as feature extractors, enabling the model to identify and understand complex relationships. When faced with new, unseen data, the model can leverage these learned features to recognize and categorize unknown events, As a result, there is a strong connection between the effectiveness of the machine learning or deep learning system and the dataset's quality. Aspects of the dataset's quality can be assessed including its size, intraclass consistency, interclass disparity, and imbalance in the distribution of the data among the many categories [8].

### 4.2 Image acquisition unit

**Fig. 8:**
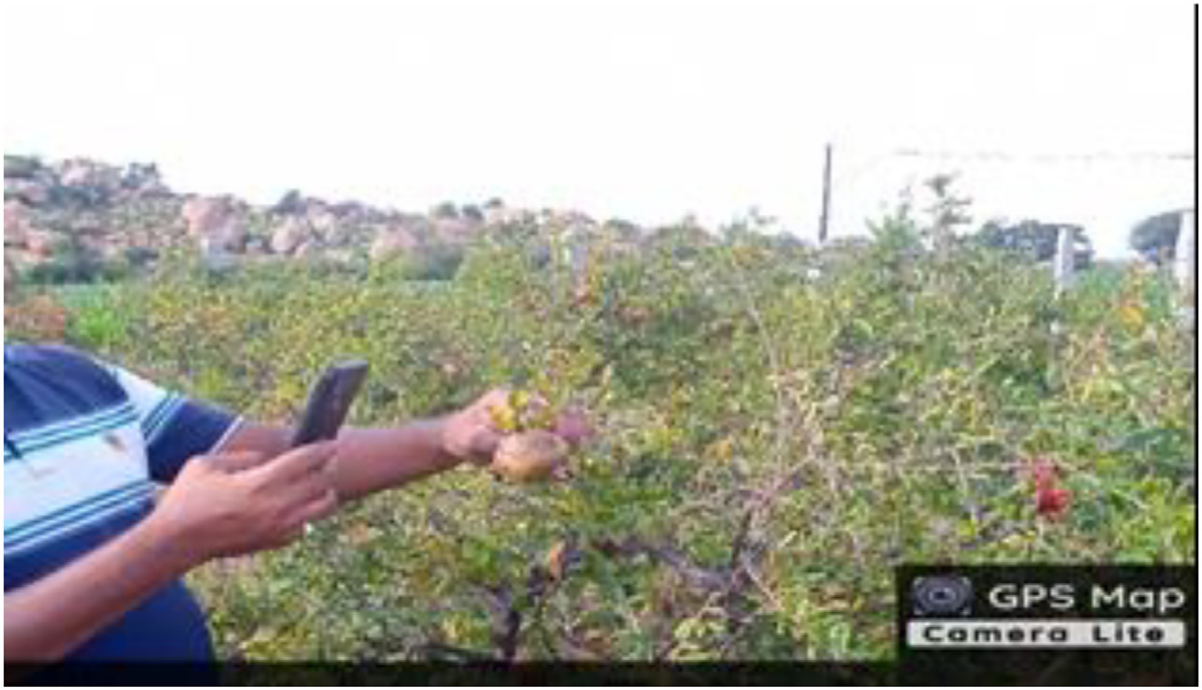
Photo taking method.

The dataset was composed using a Redmi 9 phone camera with a 13 megapixel primary sensor and a 2 megapixel depth camera f/2.2 aperture. The rear camera features a time stamp on photos, optical image stabilization, OV13B10 sensor with 1.12µm pixels, autofocus, and location information. The photos were taken with 1:1 aspect ratio, high picture quality, with different viewpoints hand-held by a person at a height of 100 cm -150 cm and a distance of 20 cm - 30 cm from the fruit. A brief description of the camera device is shown in Table 1 and the shooting method is shown in Fig. 8. Collection of pomegranate fruit diseases dataset images is shown in Table 2.

**Table 1:** Brief description of the camera device.

Sl. No.	Particulars	Description
1	Camera Maker	Xiaomi
2	Camera Model	M2006C3MII
3	Dimensions	3120 * 3120
4	F-stop	f/2.2
5	Exposure time	1/341 sec
6	ISO speed	ISO-112
7	Exposure bias	0 step
8	Focal length	3 mm
9	Flash mode	No flash
10	Metering mode	Center Weighted Average

**Table 2:** Description of the pomegranate fruit diseases dataset.

Sl. No.	Particulars	Description		
		Images count	Dimension	Resolution
1	Healthy	1450	(3120 × 3120)	2200 dpi
2	Bacterial blight	966	(3120 × 3120)	2200 dpi
3	Anthracnose	1166	(3120 × 3120)	2200 dpi
4	Cercospora	631	(3120 × 3120)	2200 dpi
5	Alternaria	886	(3120 × 3120)	2200 dpi
Total		5099		

Table 3. Summarizes the earlier work in connection with image processing of pomegranate fruits. We have searched many platforms to find a pomegranate fruit disease dataset but none of the platforms have specific pomegranate fruit disease datasets. Because the agriculture industry is inherently uncertain, creating datasets in this field is a very difficult undertaking. Due to this issue, many authors collected images locally and proceeded with their research work. Our future work will be on the pomegranate diseases dataset this dataset is extremely supportive for the machine learning or deep learning experts working in the field of precision agriculture to develop computer applications using machine learning, computer vision, and deep learning algorithms.

**Table 3:** Summary of works related to pomegranate fruits and leafs diseases detection using deep learning.

Sl. No.	References	Objective of the study	Dataset	Remarks
1	[9]	Detect and Identify Pomegranate Leaf Diseases	1844 images	Locally collected
2	[10]	Pomegranate Leaf Disease Detection	1245 images	Locally collected
3	[11]	Normal or Abnormal Pomegranate Fruit detected	6519 images	Locally collected, not classified diseases
4	[12]	Pomegranate Fruit Disease Detection	1493 fruits and leaf images	Locally collected
5	[13]	Pomegranate Fruit Disease Detection	300 images	Locally collected
6	[14]	Soybean leaf Disease Detection	1199 leaf images	Locally collected, Normal & Abnormal leaf images

## Limitations

None.

## Ethics Statement

The proposed data does not involve any human subjects, animal experiments, or data collected from social media platforms.

## CRediT authorship contribution statement

**Pakruddin B․:** Conceptualization, Formal analysis, Methodology, Investigation, Writing – original draft, Writing – review & editing. **Hemavathy R․:** Supervision, Validation, Writing – review & editing.

## Data Availability

Pomegranate Fruit Diseases Dataset for Deep Learning Models (Original data) (Mendeley Data).
